# Case Report: Diabetic Retinopathy Diagnosis Through a Mobile Screening Program

**DOI:** 10.51894/001c.165579

**Published:** 2026-07-31

**Authors:** Drishti Chauhan, C. Alex Matisse, Nisha Desai, Priyanka Bhoopathi, Rossteen Abbasi, Anthony Abro, Swati Patel, Nikhil Shirishkar, Rooz Razmi, David Goldman, Daniel Rathbun

**Affiliations:** 1 Henry Ford - Detroit

## 72

### Background

Diabetic retinopathy (DR) remains a leading cause of vision loss among working-age adults in the United States, affecting millions of individuals with diabetes. Despite established screening guidelines, adherence to annual ophthalmologic examinations remains suboptimal due to barriers such as limited access to eye care providers and socioeconomic factors. Artificial intelligence (AI)–assisted retinal screening has emerged as a potential strategy to improve access to early detection, particularly in community settings with underserved populations.

### Case Presentation

A 78-year-old female with a history of type 2 diabetes mellitus presented to a mobile, community-based DR screening event utilizing an AI-assisted fundus imaging system. The patient was asymptomatic and had not undergone ophthalmologic evaluation in over one year. Retinal imaging performed by trained volunteers and analyzed by an FDA-approved AI algorithm returned a positive screening result for diabetic retinopathy. The patient was advised to follow up with her primary care physician and ophthalmologist. Subsequent evaluation revealed mild nonproliferative diabetic retinopathy without macular edema and additional findings including posterior vitreous detachment and nuclear sclerosis. The patient was managed conservatively with regular monitoring and improved glycemic control, with no evidence of disease progression at follow-up.

### Conclusion

This case highlights the potential utility of AI-assisted diabetic retinopathy screening in community-based settings to identify undiagnosed disease and facilitate timely referral for ophthalmologic care. Mobile screening programs leveraging AI technology may improve early detection of DR and help address disparities in access to eye care, particularly among high-risk and underserved populations.

